# HCG Trigger After Failed GnRH Agonist Trigger Resulted in Two Consecutive Live Births: A Case Report

**DOI:** 10.3389/frph.2021.764299

**Published:** 2021-10-27

**Authors:** Sara Liest, Iben Riishede Christiansen, Lisbeth Prætorius, Jeanette Bogstad, Nina la Cour Freiesleben, Anja Pinborg, Kristine Løssl

**Affiliations:** ^1^Department of Obstetrics and Gynecology, North Zealand Hospital, Hillerød, Denmark; ^2^Center of Fetal Medicine and Pregnancy, Department of Obstetrics, Copenhagen University Hospital Rigshospitalet, Copenhagen, Denmark; ^3^Institute of Clinical Medicine, Faculty of Health and Medical Sciences, University of Copenhagen, Copenhagen, Denmark; ^4^The Fertility Clinic, Department of Obstetrics and Gynecology, Copenhagen University Hospital Hvidovre, Hvidovre, Denmark; ^5^The Fertility Clinic, Copenhagen University Hospital Rigshospitalet, Copenhagen, Denmark

**Keywords:** empty follicle syndrome (EFS), rescue hCG, failed agonist trigger, re-trigger with hCG, consecutive live births after empty follicle syndrome

## Abstract

**Background:** Failed gonadotropin-releasing hormone (GnRH) agonist trigger with no oocyte retrieved during aspiration of several follicles is a rare but recurrent situation that can be rescued by the termination of the aspiration procedure, retriggering by human chorion gonadotropin (hCG), and repeated oocyte pickup 36 h later. Failed GnRH agonist trigger is frustrating and unsatisfactory, and fertility doctors must be aware of possible hCG retriggering and retained opportunity for successful cycle outcome.

**Objective:** In this case report, we present a woman who experienced failed GnRH agonist trigger and rescue hCG retrigger followed by two consecutive live births after frozen-thawed single blastocyst transfers.

**Methods:** A case report.

**Results:** Two healthy children were born in 2018 and 2020, respectively as a result of controlled ovarian stimulation for IVF, failed GnRH agonist trigger followed by hCG re-trigger, and successful retrieval of 25 oocytes.

**Conclusion:** Retriggering with hCG after failed GnRH agonist trigger can result in consecutive live births, and such knowledge can prevent cycle cancellation and patient discouragement. Knowledge on retriggering with hCG and consecutive live births after failed GnRH agonist trigger can prevent cycle cancellation and patient discouragement.

## Introduction

Ovarian hyperstimulation syndrome (OHSS) is a serious complication to *in vitro* fertilization (IVF), which is potentially life-threatening. Moderate to severe OHSS has a prevalence of 3–8% (1, 2) being highest for women with polycystic ovarian syndrome (PCOS). The risk of OHSS increases with an increasing number of growing follicles and also high oestradiol levels on the day of ovulation trigger, and it increases with an increased number of retrieved oocytes (3, 4). More than 18 growing follicles ≥11 mm on the day of ovulation trigger is a good predictor of severe OHSS (5, 6). The live birth rate increases with number of oocytes retrieved up to 15 in fresh IVF cycles and then plateaued (4), whereas the cumulative live birth rate steadily increases with number of oocytes reaching 70% when 25 oocytes were retrieved (7).

In the GnRH antagonist protocol, the risk of OHSS is significantly reduced when final oocyte maturation is triggered by a GnRH-agonist (GnRHa), which stimulates the endogenous pituitary secretion of luteinizing hormone (LH), instead of the traditionally used human chorionic gonadotropin (hCG) trigger (8). hCG provides LH activity with a much longer half-life than endogenous LH and promotes the production of vascular endothelial growth factor (VEGF) by endothelial cells (9), which is thought to be the principal vasoactive mediator causing the increased vascular permeability seen in OHSS.

The GnRHa trigger has been introduced as the trigger of choice in women at risk of OHSS, which is easily judged based on the number of growing follicles on trigger day during the standard transvaginal ultrasound examination that is always performed during IVF. The use of GnRHa trigger does not hamper the yield of mature oocytes (10). Another strategy used to reduce the risk of OHSS is to freeze all developed embryos to avoid pregnancy-related endogenous hCG production. Postponed embryo transfer to a subsequent natural or artificial frozen-thawed embryo transfer does not decrease the chance of ongoing pregnancy (11–13).

The administration of a GnRHa initiates the endogenous surge of LH that is mandatory for the final oocyte maturation proceeding ovulation. However, a suboptimal LH response can reduce or inhibit oocyte yield during oocyte retrieval (14). Failed GnRHa trigger, also called empty follicle syndrome (EFS), is defined as the failure to retrieve oocytes from preovulatory follicles under oocyte pickup despite flushing. EFS has been reported with an incidence ranging from 1.0% to 3.5% after the use of GnRHa trigger (15–17). The use of hCG as a rescue trigger administered 36 h before a second oocyte retrieval has been described in other cases (18, 19), but might not be common knowledge among all the doctors involved in fertility treatment.

The present case is important to provide knowledge about the possibility of consecutive live births even in cycles with failed GnRHa trigger, if handled properly. Correct counseling is very helpful in clinical situations where this unexpected situation appears. To the best of our knowledge, two consecutive live births as a result of hCG retrigger have not previously been reported.

Informed written consent has been obtained from the woman for publication of the case report.

## Case Description

In 2016, a 29-year old, nulligravida, healthy woman and her partner were referred to a tertiary clinic because of combined infertility and unsuccessful intrauterine inseminations. The duration of infertility was 1 year. The female diagnosis was PCOS based on secondary amenorrhea and an antral follicle count of 80 corresponding to an antimüllarian hormone (AMH) level of 96 pmol/L. Androgen status, prolactin, and thyroid stimulating hormone levels were normal. Follicle stimulating hormone (FSH) and LH levels were 5.8 IU/L and LH 6.7 IU/L, respectively. BMI was 21 kg/m^2^. The sperm volume and count were just within the normal range, but the motility was affected with a lower than a normal number of progressively motile sperm cells according to the recommendation of ICSI.

The woman underwent a total of three controlled ovarian stimulation cycles that are summarized in Table 1. All three were in a flexible GnRH antagonist protocol. In the first two stimulations, final oocyte maturation was triggered with hCG as the risk of OHSS was not pending based on the number of follicles > 11 mm. After the first ovarian stimulation 11 oocytes were retrieved, but none of them developed into blastocysts. In the second stimulation, 12 oocytes were retrieved, one cleavage stage embryo was transferred, and the last embryos did not develop into blastocysts. None of the first two simulations resulted in pregnancy.

**Table 1:** Overview of the three controlled ovarian stimulation cycles and associated outcome.

**Treatment number (date of oocyte retrievals)**	**1. (16/11 2016)**	**2. (15/2 2017)**	**3. (1/4+/3/4 2017)**
Days of stimulation	21	31	15
Gonadotropin starting dose	Menopur 75 IU	Puregon 91 IU	Menopur 112 IU
Gonadotropin total dose	1,872 IU	3,295 IU	2,051 IU
Fertilization method	ICSI	IVF/ICSI 6/6 oocytes	ICSI
Number of oocytes	11	12	(0)/25^*^
Number of mature oocytes	5	NA/3	20
Number of cleavage stage embryos	4	0/2	13
Number of blastocysts^**^	0	0	8
Embryos transferred in the stimulated cycle	0	1	0
Number of FET cycles	0	0	7^***^
Pregnancies	0	0	2
**Live births**	**0**	**0**	**2 (2018** **+** **2020)**

* *Zero oocytes were retrieved after GnRHa trigger, after rescue hCG re-trigger 25 oocytes were retrieved*.

** *Gardner score (on Day 5 or 6) ≥ 3BB*.

*** *One blastocyst underwent atresia after warming*.

In the third ovarian stimulation, a daily dose of human menopausal gonadotropin (Menopur; Ferring Pharmaceuticals) 112–131 IU was administered from cycle day 2 (CD2), and 0.25 mg of GnRH antagonist (Cetrotide; Merck) was added daily when the leading follicles reached 13–14 mm (stimulation day 12). On stimulation day 16, when ~15 follicles reached 17 mm, and a total of 23 follicles were ≥11 mm, the final oocyte maturation was triggered with 0.50 mg of GnRH agonist Suprefact (Buserelin; Orifarm). Elective freeze-all was planned to reduce the risk of OHSS. Oocyte retrieval was performed 36 h after trigger using ultrasound-guided transvaginal needle aspiration after administration of local anesthetic and low-dose intravenous opioids. Approximately 12 follicles from the right ovary were emptied without retrieval of any oocytes, even though flushing was used in several follicles. The procedure was stopped, and five follicles in the right ovary and all the follicles in the left ovary were left untouched. No GnRH antagonist was administered after the oocyte retrieval. Rescue hCG retrigger was performed at 10 pm the same day with chorion gonadotropin-alfa (Ovitrelle; Merck) 250 μg, and 36 h later a second oocyte retrieval was performed. Here 25 oocytes, of which 20 were mature, were retrieved from 30 follicles, 13 were fertilized and five-six days after the second oocyte retrieval, eight good quality blastocysts were vitrified. There were no symptoms of OHSS after rescue hCG retrigger.

The patient used contraceptive pills for 1 month and consecutive frozen-thawed embryo transfer (FET) was planned in artificial hormone replacement cycles (AC). In the fourth AC FET the patient conceived and delivered a healthy girl at gestational age 40+0 weeks, 4 days after initiation of partus provocatus medicamentalis by oral misoprostol. The weight of the girl was 2,155 g (−34%). In the AC FET, the patient took 4 mg oestradiol twice daily for 17 days before the addition of 90 mg of vaginal progesterone gel (Crinone; Orifarm) daily. Single blastocyst warming and transfer were performed on the 6th day of progesterone.

After 18 months, the couple returned with the wish for a second child. There were still four vitrified blastocysts left. In the third and last AC FET cycle (one blastocyst did not survive warming), the patient conceived again and delivered a healthy boy at gestational age 40+0 weeks, weight 2,900 g and length 52 cm. In the second pregnancy, the patient was treated with acetylsalicylic acid 150 mg daily from gestational age 12 weeks because of IUGR in first pregnancy.

In our case, rescue hCG retrigger, given after a failed GnRHa trigger and EFS, and a second oocyte retrieval turned out to be a successful treatment with 25 oocytes retrieved, eight blastocysts suitable for vitrification, and finally two pregnancies and live births.

## Discussion

To our knowledge, we report for the first time a case of failed GnRHa trigger that ended up as a successful ICSI treatment with two consecutive live births as a result of rescue hCG re trigger. This was possible due to awareness of the phenomenon and interruption of the oocyte retrieval procedure in due time to perform hCG retrigger, followed by a second oocyte retrieval. The case is important in order to provide knowledge about consecutive live births even in cycles with failed GnRHa trigger if handled properly. Correct counseling from clinicians to patients is needed in clinical situations where this unexpected situation appears.

Several risk factors of failed agonist trigger have been identified: low BMI, low baseline LH, high total dose of administered gonadotropins, hypogonadotropic hypogonadism, or iatrogenic-induced pituitary downregulation in the long GnRHa protocol or after prolonged use of oral contraceptives (20). Patients with BMI <22 kg/m^2^ have a twice as high failure rate as patients with higher BMI (*P* = 0.039), baseline LH <2 IU/L has a statistically significant higher failure rate compared to patients with a baseline LH ≥ 2 IU/L (5.6 vs. 1.8%, *P* = 0.048), and patients who received >3,800 IU gonadotropins during stimulation had a 3.5 times higher failure rate compared with patients receiving <3,800 IU (*P* < 0.0001) (19).

In the current case, there were no issues regarding patient compliance, and most likely the reason for failed GnRHa trigger was a suboptimal endogenous LH surge. Even though gonadotropin levels were measured and found within the mid-normal range as a part of the standard fertility work-up of this anovulatory women (LH 6.7 IU/L), no LH measurement was performed at the initiation (baseline) of this third ICSI treatment, which is a limitation to causal interpretation. However, the patient did use oral contraceptive pills (OCP) to initiate her bleeding, and the baseline LH level may have been suppressed.

As the incidence of EFS is generally low (15–17), and the sensitivity of baseline LH levels to predict EFS is low (15), we would suggest measurement of baseline LH level “on indication” rather than on all patients. Such indications could be the use of OCP or other drugs that induce pituitary downregulation even after a wash-out period and/or anovulation, especially if the anovulation is not a part of a PCOS diagnosis where LH levels are usually normal–high. If baseline LH is low after OCP, postponement of stimulation start could be applied if only small antral follicles (proceeded by another LH measurement), or a cautious gonadotropin dose for ovarian stimulation could be chosen aiming for hCG trigger with a minimal risk of OHSS.

In the present case, supplementary GnRH antagonist was not used after the first failed oocyte retrieval. We would not expect premature ovulation to be an issue in cases of genuine EFS. The initial GnRHa trigger is expected to empty the pituitary LH, and if this endogenous LH should reach a circulatory “threshold level” of concern, we would expect a suboptimal response with at least a few oocytes retrieved at the first retrieval rather than zero oocytes (genuine EFS).

Another question is whether to measure the LH level in blood or urine in the morning after the GnRHa trigger. Interestingly, it has recently been demonstrated that a standard urinary LH test on the morning after GnRHa trigger can be used by patients at home as an easy and convenient way to document a sufficient LH release in response to GnRHa trigger (21). Only three out of 359 (0.8%) urine LH tests were negative; however, in one out of the three cases, an LH measurement in blood showed LH rise consistent with optimal response to the GnRHa trigger (21). Thus, the predictive value of a negative urine LH test does not seem reliable and could have resulted in ovulation if hCG retriggering and rescheduling of the oocyte retrieval had been based on the urine LH testing alone.

In our opinion, insufficient LH rise after GnRHa trigger is too uncommon to justify posttrigger LH measurements. Instead, if no oocytes are retrieved after proper emptying of ~7 preovulatory follicles, we advocate hCG retrigger and a second oocyte retrieval, as presented in this case report.

The diagnosis EFS could be given when no oocytes are retrieved from ~7 preovulatory follicles despite proper emptying and flushing. The risk of OHSS has been suggested to be reduced after failed GnRHa and hCG retrigger due to reduction of the oestradiol level after the first attempt to retrieve oocytes (18) as well as by an elective freeze-all regimen. Moderate to severe OHSS after rescue hCG retrigger has nevertheless been described, and an individual estimation of OHSS risk before hCGretrigger is important in each patient (19, 22). In this case report, we lack data on posttrigger serum LH, progesterone, and oestradiol.

The experience from the presented case report is that rescue hCG retrigger after failed GnRHa trigger can result in a complete family formation. The case is important as knowledge on retriggering with hCG and consecutive live births after failed GnRHa can prevent cycle cancellation and patient discouragement. Correct counseling from clinicians to patients is needed in clinical situations where this unexpected situation appears.

Perspective of the patient: Sharing the knowledge that hCG retrigger after failed GnRHa trigger is possible can prevent cycle cancellation and patient discouragement and result in consecutive live births.

## Data Availability Statement

The original contributions presented in the study are included in the article/supplementary material, further inquiries can be directed to the corresponding author/s.

## Ethics Statement

Informed written consent has been obtained from the woman for publication of the case report.

## Author Contributions

IR, LP, JB, NF, AP, and KL helping with litterature, references, and reading correture on article. All authors contributed to the article and approved the submitted version.

## Conflict of Interest

The authors declare that the research was conducted in the absence of any commercial or financial relationships that could be construed as a potential conflict of interest.

## Publisher's Note

All claims expressed in this article are solely those of the authors and do not necessarily represent those of their affiliated organizations, or those of the publisher, the editors and the reviewers. Any product that may be evaluated in this article, or claim that may be made by its manufacturer, is not guaranteed or endorsed by the publisher.
